# Concurrent Odontogenic Keratocyst and Odontoma: Report of an Unusual and Rare Entity

**DOI:** 10.30476/dentjods.2023.98278.2066

**Published:** 2023-12-01

**Authors:** Fatemeh Akbarizadeh, Javad Garmabi, Maryam Paknahad

**Affiliations:** 1 Oral and Dental Disease Research Center, Oral and Maxillofacial Radiology, School of Dentistry, Shiraz University of Medical Sciences, Shiraz, Iran; 2 Postgraduate Student, Oral and Maxillofacial Radiology, School of Dentistry, Shiraz University of Medical Sciences, Shiraz, Iran

**Keywords:** Odontogenic Cysts, Odontoma, Radiography, Cone-Beam Computed Tomography

## Abstract

Hybrid lesions of jaws are rare entities defined as two different lesions co-occurring in the same location, with identical histopathological origin. Ameloblastoma, calcifying cystic odontogenic tumor and odontoma are among the most common lesions that have been reported to combine with other lesions. In this study, a hybrid lesion of odontogenic keratocyst (OKC) and odontoma in the mandible of a forty-five years old male reported. Additional to the rarity of this hybrid lesion, the present case had unique radiologic features, including atypical location and extension of the lesion and profound knife-edge root resorption of the teeth in the area, which was not a common feature for any of the two lesions. The surgical procedure was marsupialization to reduce the size of the lesion. As a result of the surgery, the healing of the surgical wound was uneventful. In addition, careful follow-up for the patient was conducted, which had no recurrence till now (after 15 months).

## Introduction

The odontogenic keratocyst (OKC) is a benign but aggressive intraosseous tumor drawn from the remains of the original tooth germ or dental lamina [ 1 ]. OKC can develop in association with an unerupted tooth or as a solitary entity in bone [ 2 ]. This cyst demonstrates a propensity for aggressive behavior and a relatively high recurrence rate compared to the other odontogenic cysts [ 1 ]. Ameloblastomatous change and malignant transformation of this cyst were also reported [ 3 ].

OKCs are classified as central (intraosseous) versus peripheral or mucosal forms. The central form is further subclassified as parakeratotic, orthokeratotic, or mixed [ 4 ]. There is a predilection for posterior jaws and the mandible is affected two to four times more often than the maxilla [ 5 ]. Clinically, OKCs usually cause no symptoms and manifestations, although mild swelling may occur and is the most common clinical manifestation [ 1 ]. Pain may also occur in case of secondary infection [ 5 ]. Radiographically, they appear as unilocular or multilocular radiolucencies with a corticated or sclerotic, reactive bony rim. The margin of the lesion may be smooth or scalloped. Sometimes, it may have destructive borders, invading the adjacent structures [ 6 ].

Odontomas are the most prevalent odontogenic hamartoma of the jaws, with a frequency of 35%–76%, characterized by their non-aggressive behavior [ 7 ]. These tumors produce both the epithelial and mesenchymal components of the dental apparatus with complete, mature differentiation. They produce Enamel, dentin, cementum, and pulp [ 8 ]. Odontoma is classified into two main types including compound and complex. The more common type, compound odontoma, is consisted of several tooth particles, while the complex type is a heterogeneous mass of dental tissue [ 9 ]. The predominant location for odontoma is anterior of the maxilla and posterior of the mandible in compound and complex odontoma, respectively [ 7 ]. 

Co-occurrence of two different lesions in the jaw is categorized into two types including collisional and hybrid lesions. The collisional lesions are tumors with different histological origins, which exclusively co-exist in the same region. However, the hybrid lesions, which are rare, are diverse tumoral demonstrations of the same histopathological source [ 9 ]. 

Extensive lesions in the jaws need to be completely investigated to make a distinction between their less aggressive compartment and the threatening compartment, so as to choose an appropriate surgical approach for their treatment. The treatment plan and the prognosis of OKC and odontoma are completely different. Therefore, precise diagnosis of the hybrid lesion is necessary.

To the best of our knowledge so far, only two cases have reported the co-occurrence of OKC and odontoma [ 6 , 10 ], therefore the present case would be the third case reporting of such co-occurrence in the literature. 

## Case Presentation

**Figure 1:**
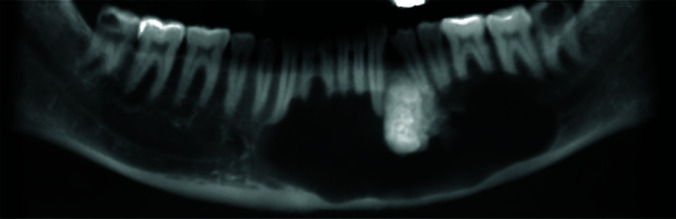
The reconstructed panoramic view showing the extension of the lesion and the radiopacity is also clearly detected

A 45-year-old male was referred to the Oral and Maxillofacial Radiology Department by a surgeon. The patient had attended dental school to fill his left maxillary tooth. After taking a panoramic radiograph, a mixed radiopaque-radiolucent lesion was detected incidentally in the mandible. The patient had no history of pain/ tenderness or previous surgical manipulation in his mandible. Also, he had no systemic disease. However, the intraoral clinical examination revealed a mild swelling on the left side of the buccal and palatal cortices. Cone-beam computed tomography of the mandible was prescribed for the patient to establish a thorough radiographic assessment. The cone-beam computed tomography images demonstrated a large mixed radiolucent-radiopaque lesion. The radiolucent compartment had extended in the periapical area of mandibular teeth from the mesial aspect of the left second molar tooth to the distal part of the right canine, crossing the midline. In addition, root divergence of the canine and first premolar teeth in the left side was noticed. A non-homogenous radiopaque mass with denticle-like density was detected interdentally between the canine and the first premolar teeth on the left side (Figure 1). The superior border of the lesion was scalloping between the teeth, especially on the right side.

**Figure 2:**
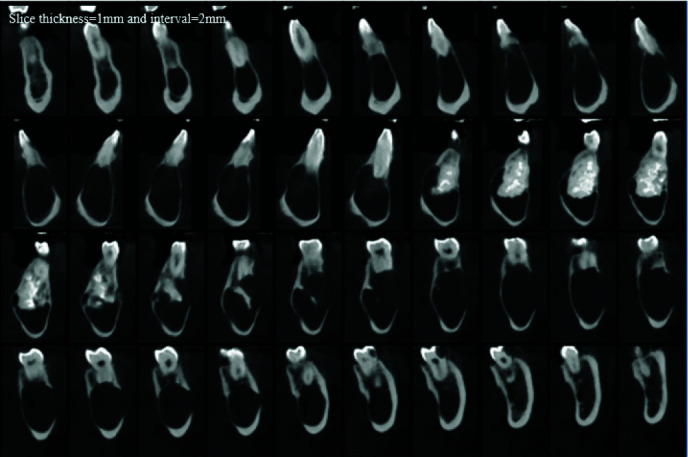
Cone beam computed tomography scan shows thinning of both buccal and lingual cortices and loss of continuity of buccal cortex

**Figure 3:**
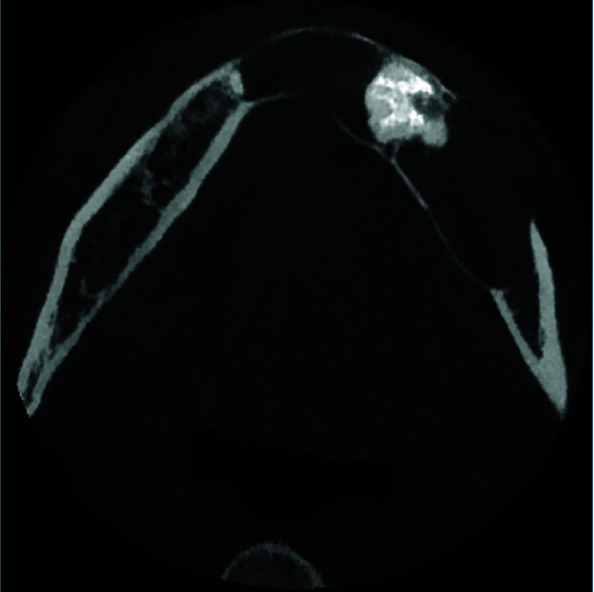
Axial image shows the expansion of the lesion, discontinuity of the buccal cortex, thinning of both cortices and radiopacity of complex odontoma

**Figure 4:**
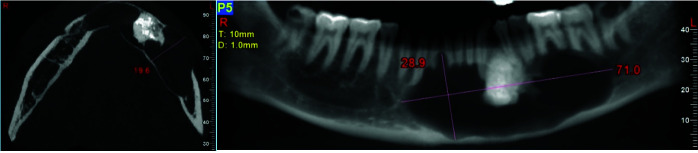
Dimensions of the lesions in axial and reconstructed panoramic images

Extreme knife-edge root resorption in all involved teeth in the lesion was evident. Thinning, expansion and loss of continuity of both buccal and lingual cortices, thinning and endosteal erosion of the mandible's inferior border were also detected. (Figures 2-4) Moreover, involvement and displacement of the inferior alveolar nerve canal, resulting in loss of cortical boundaries was noted and mental foramen could not be followed.

An incisional biopsy of the mandible was carried out under general anesthesia. The specimen received in formalin solution and consisted two separate bottles.

The first bottle contained multiple pieces of irregular creamy-brown elastic tissues and the second bottle contained three pieces of irregular creamy bony hard tissues. The histopathological examination revealed odontogenic epithelium and basophilic enamel matrix. They also disclosed the cystic space-lined connective layer of stratified epithelium with a prominent basal cell layer and the absence of rete ridges. The connective tissue showed an inflammatory response. Eosin-stained odontogenic components with adjacent clear spaces were visible, composed of dental hard tissue and mesenchyme distributed in fibrous tissue.

**Figure 5:**
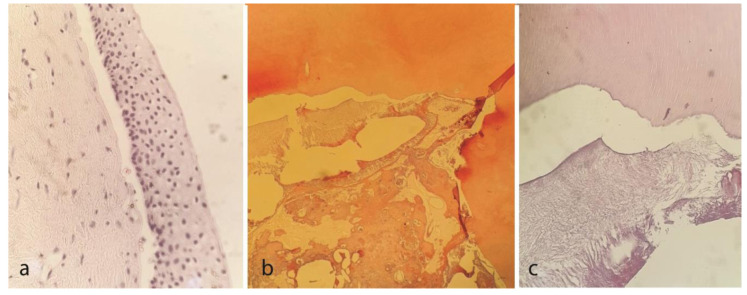
Images were taken at magnification of 200× (A), 100× (B), 200× (C) Eosin-stained odontogenic components with adjacent clear spaces, **a:** Dental hard tissue and mesenchyme, distributed in fibrous tissue, **b:** Mature tubular dentin with incremental lines, **c:** Randomly placed unstructured eosin-stained hard tissue component and cementum like structures in periphery

Mature tubular dentin with incremental lines randomly placed unstructured sheets of eosin-stained hard tissue component, and cementum-like structures in the periphery were seen. A thorough histopathological examination diagnosed an inflamed OKC associated with complex odontoma (Figure 5).

**Figure 6:**
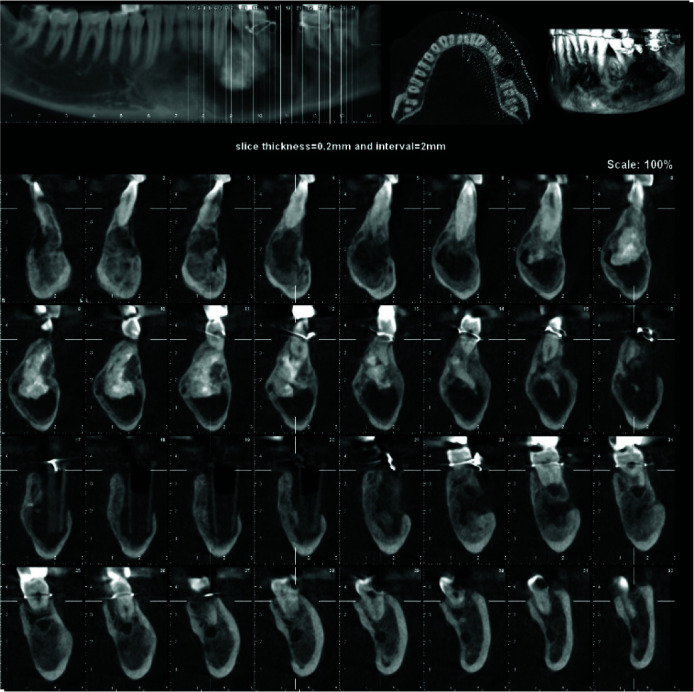
The follow up CBCT image after 15 months illustrated healing and reduction of size of the lesion

The surgical procedure was marsupialization to reduce the size of the lesion. As a result of the surgery, the healing of the surgical wound was uneventful (Figure 6). The patient was kept under observation at regular intervals to detect any recurrence. 

## Discussion

OKC is a common developmental cyst, possibly originating from remnants of epithelium, whether from tooth germ or dental lamina. Although in previous classifications of WHO, OKC was considered to have a dual manner as a cyst or tumor, in the most recent category, it is exclusively viewed as a cyst [ 1 ]. This cyst has aggressive behavior and a high recurrence rate (%5-%62.5) [ 1 ]. In addition, ameloblastomatous change and malignant transformation of OKC are possible [ 3 ]. Several hybrid lesions have been reported to date.

Ameloblastoma, calcifying cystic odontogenic tumor and odontoma are among the most common lesions that have been reported to combine with other lesions [ 6 , 9 , 11 ]. The most common lesions that have been reported to co-occur with odontoma are calcifying odontogenic cyst, adenomatoid odontogenic tumor, ameloblastic fibroma and ameloblastic fibro-odontoma [ 9 , 12 - 13 ]. Limited reports of combined odontoma with botryoid odontogenic cyst, glandular odontogenic cyst, and cemento-ossifying fibroma were also found [ 9 , 14 - 15 ].

To the best of our knowledge, there were only two reports of combined OKC with odontoma in literature till now. Bang *et al*. [ 10 ] reported a case of combined OKC, which interestingly co-occurred with erupting odontoma. Moreover, Kulkarni *et al*. [ 6 ] reported an orthokeratinized odontogenic cyst with complex odontoma in the maxilla, which has caused suffering in the patient's breathing. 

The co-occurrence of OKC with odontoma is rare and worthy of attention. Additionally, our present case was unique regarding its location. The extension of OKC in the present case was predominantly in the anterior mandible crossing the midline, while OKC is mainly found in the posterior of the mandible [ 1 ]. Also, the accompanying complex odontoma was located inter-dentally between the roots of the anterior teeth, which is an atypical location for a complex odontoma. The other unusual feature of the present case was profound, knife-edge resorption of the teeth in the region. The study conducted by Kitisubkanchana *et al*. [ 11 ] revealed that the frequency of root resorption in OKC was %7 and OKC usually causes mild or no root resorption. The possible justification for these divergent features is the pluripotentiality of the odontogenic epithelium, leading to unusual odontogenic lesions' behaviors [ 3 ].

A clinical point worth mentioning is that if the treatment plan for teeth involved by OKC is extraction, the surgical procedure should be conducted as atraumatic as possible to prevent the cyst's rupture, because injury to the cyst and its rupture leads to the superimposition of infection and systemic symptoms in the patient [ 1 ]. However, in the present case, the teeth were kept.

The high recurrence rate, and ameloblastomatous, and malignant transformations of OKC impose the surgeon to conduct a thorough presurgical assessment of this cyst. In hybrid cases of OKC, the evaluation should not be underemphasized due to the other less aggressive compartment, which was a complex odontoma in the present case. Moreover, conservative marsupialization of this cyst is the least aggressive surgical treatment for this lesion, which was conducted, in the present case.

A written consent was obtained from the patient before imaging to use the patient’s images in research works.

## Conclusion

Extensive lesions in the jaws need to be completely evaluated to differentiate between their minimally invasive compartment and the threatening one, in order to select the appropriate surgical approach for surgeons. The current lesion is a rare case of an extensive OKC associated with a complex odontoma for which, marsupialization was performed due to the large size of the lesion. Healing was uneventful and showed bone regeneration in the surgical defect. The patient has been followed up for over 15 months and no signs of recurrence have been detected so far. 
